# Study of Fermentation Strategies by *Lactobacillus gasseri* for the Production of Probiotic Food Using Passion Fruit Juice Combined with Green Tea as Raw Material

**DOI:** 10.3390/foods11101471

**Published:** 2022-05-18

**Authors:** Wanessa Dayane Leite Lima, Shênia Santos Monteiro, Matheus Augusto de Bittencourt Pasquali

**Affiliations:** 1 Department of Food Engineering, Federal University of Campina Grande, Campina Grande 58428-830, PB, Brazil; wanessadayane@gmail.com; 2 Center for Technology and Natural Resources, Federal University of Campina Grande, Campina Grande 58428-830, PB, Brazil; shenia-monteiro@hotmail.com

**Keywords:** *Camellia sinensis*, *Passiflora edulis*, probiotic

## Abstract

Foods fermented by *Lactobacillus* with probiotic properties convey health benefits to consumers, in addition to fulfilling the basic function of nourishing. This work aimed to evaluate the growth characteristics of *L. gasseri* in passion fruit juice and passion fruit added with green tea. Fermentation under evaluation of different pH (3.5–7.5), temperature (30–44 °C), and with the addition of green tea (7.5–15%), took place for 48 h. The results showed that a pH of 7.5 and temperature of 44 °C showed higher cell production, and it was also verified that the addition of 15% of green tea induced the growth of *L. gasseri* in passion fruit juice. The concentrations of probiotic cells observed were above 9 Log CFU.mL^−1^ and, therefore, they are promising products for consumption as a functional food and application in the food industry with potential health benefits.

## 1. Introduction

Due to the presence of microorganisms, fermented foods have unique properties, including probiotics, antimicrobials, antioxidants, peptides, and other compounds that, when consumed in adequate proportions, convey benefits to the health of consumers [1]. The enormous relevance currently devoted to the importance of the gut microbiota in human health and well-being has led to increased interest in the development of new probiotic delivery systems [2]. Although dairy is traditionally considered the best food matrix for probiotics, there are some disadvantages related to the composition of milk, such as hypersensitivities, and in addition an estimated 600 million people worldwide are vegetarian [3]. These are some of the reasons why many studies are being conducted to study plant-based matrices for the development of innovative and attractive probiotic foods.

Each food matrix has unique properties and advantages that may favor the aggregation of probiotics in foods, but may also impose technological barriers [4]. Researchers have focused on the use of fruit and vegetable juices for fermentation by lactic acid bacteria to provide functional foods as an alternative for individuals who cannot consume dairy products [5,6,7]. Fruit juices have been reported as a novel and appropriate medium for microorganisms, combining nutritional effects with the value-added benefits of probiotics [4]. In addition, fruit juices have shown action against pathogenic microorganisms, while improving the growth of probiotics, due to the content of bioactive and essential nutrients [8].

Passion fruit juice (*Passiflora edulis*) has nutritional properties which have been reported in the literature, emphasizing the content of total carotenoids, total flavonoids, and polyphenols [4]. From the genus *Passiflora*, around 600 species worldwide have been described prior to the present study, more than half found in the American territory, with economic importance in countries such as Ecuador, Colombia, Peru, Indonesia and Kenya [9], 120 species are native to Brazil [10], and it is widely cultivated in tropical areas [11]. In Brazil, the *Passiflora edulis* fruit is responsible for 95% of the cultivated area [12]. Relevant biological effects are attributed to passion fruit’s antioxidant [13], antihypertensive [14], anti-inflammatory [15], and anxiolytic actions [16]. In addition to the medicinal properties of the fruit due to its protective effect in preventing diseases, bringing several benefits to consumer health, passion fruit contains several aromatic substances and organic acids, suitable for processing juice with a delicious and fragrant flavor [11,17].

In previous studies, passion fruit was used as a carrier matrix for probiotics. Farias et al. [18] showed that the fermented juice has advantages over the unfermented juice, showing viability of *Lactobacillus rhamnosus* above 8 Log after 28 days of refrigerated storage; in addition, the juice also contains lactic acid, a natural antibiotic. Passion fruit varieties, such as passion fruit from the caatinga, also showed potential for use in kefir production [19].

Like passion fruit, green tea leaves (*Camellia sinensis* L.) contain polysaccharides, polyphenols, amino acids, proteins, superoxide dismutase, catalase, caffeine, vitamins, and other nutrients that have been proven to have various functions such as antioxidant, antidiabetic and hypoglycemic activity [20,21]. In addition, bioactive compounds are associated with beneficial changes in the gut microbiota. Studies such as the one carried out by Zhang et al. [22] show the beneficial effects on the intestinal microbiota of anthocyanins from purple sweet potato, which was observed to favor the development of probiotics and the inhibition of pathogenic microorganisms. Furthermore, Wang et al. [23] showed that fermenting green tea infusion with probiotics would be an innovative way to modulate tea flavor and develop new products with high probiotic counts. In addition to these studies, Wang et al. [24] showed that green tea fermented by lactic acid bacteria can act as a functional ingredient to contribute to anti-obesity effects. However, it is observed in these studies that the composition of the culture medium, the fermentation conditions and the strain used in the process influence the product, so that it is essential to investigate the effects of different raw materials and process conditions for the optimization and development of new probiotic foods. These are some of the factors that led to the objectives of this work, which are defined below.

The first objective was to know the characteristics of the fermentation kinetics of media formulated with passion fruit and passion fruit added with green tea by *L. gasseri* under different process conditions. *L. gasseri* has significant potential for application as a probiotic as it meets certain criteria: being of human origin, non-pathogenic, non-toxigenic, non-invasive, devoid of transmissible antibiotic resistance genes, resistance to technological processes, demonstrating acid and bile tolerance, adhesion to epithelial tissue, transient persistence in the gastrointestinal tract, produces antimicrobial substances, antagonizes pathogens and prevents infections, modulates immune responses and positively influences metabolic activity [25,26]. The second objective was to evaluate the influence of the aggregation of green tea infusion in passion fruit juice on the growth of the probiotic culture in the fermentation medium.

## 2. Materials and Methods

### 2.1. Raw Material

The basis for this study was passion fruit (*Passiflora edulis*) juice. To obtain this, ripe fruits were purchased at the local market in the city of Campina Grande, PB. Fruits with completely yellow skin and smooth surface were sanitized, washed in running water to remove residues from the field, and sanitized in a chlorinated solution at 200 ppm of active chlorine for 15 min. Then, the fruits were submitted to pulp removal, the pulp together with the seed passed through sieves with 2.5 mm mesh for separation, then stored at −20 °C.

To study the relationship between the formulation of the raw material and the growth of *Lactobacillus gasseri* BNR17 (*L. gasseri*) acquired lyophilized (Farmar Oficial, São Roque, Brazil), formulations with passion fruit supplemented with green tea (*Camellia sinensis* L.) were evaluated. Green tea was purchased in the form of crushed dry leaves, of the Maratá brand. An infusion was prepared with the green tea plant material, using 10 g to 150 mL of distilled water. The infusion was prepared at 70 °C under constant stirring for 15 min. The mixture was filtered and stored at −20 °C.

### 2.2. Fermentation Process Design

Two experimental matrices were set up to study the ideal conditions for the fermentation process. Complete experimental design plays a key role in screening the multiple variables. The first experimental matrix was designed to study passion fruit juice as the main raw material, using the 2² complete experimental design with three repetitions of the central point. The independent variables evaluated in the process of fermentation of passion fruit juice by *L. gasseri* were pH (3.5–7.5) and incubation temperature (30–44 °C).

The second experimental matrix was designed to study the effect of adding green tea to the formulation of raw material for fermentation by *L. gasseri*. In this experimental design, a 2³ matrix with three repetitions of the central point was adopted. The independent variables were studied were pH (3.5–7.5), temperature (30–44 °C), and concentration of green tea infusion (7.5–15%).

The pH and temperature ranges were selected taking the center of the levels of the independent variables, the conditions widely reported in the literature for the growth of bacteria of the genus *Lactobacillus* (pH 3.5 and temperature of 37 °C), by simulating the conditions during digestion in the stomach. and or in the presence of bile salts in the small intestine [27]. The formulations in each process condition are described in Table 1.

### 2.3. Inoculation and Preparation of the Fermentation Medium

The passion fruit juice pH was adjusted to 3.5, 5.5, and 7.5 with a 4 M NaOH solution. The concentration of green tea infusion was added at concentrations of 7.5%, 11.3%, and 15%, and the pH was adjusted. Heat treatment was applied to the different formulations of the fermentation medium, where the media were subjected to heating at 82 °C for 20 s, as described by Sanchez et al. [28].

For inoculation of the probiotic culture in the fermentation media, methods were studied for viability analysis, as described by Lin et al. [29] However, based on previous tests (unpublished data), the methodology described below was adopted because it presents advantages such as obtaining rapid results, and presenting results comparable to other methods described in the literature. Therefore, the lyophilized culture of *L. gasseri* was inoculated into the medium cooled at temperatures of 30 °C, 37 °C and 44 °C, at a rate of 9 Log CFU for 1 mL of the mixture with passion fruit and homogenized for 10 min. Once the lyophilized culture was inoculated directly into the fermentation medium, the initial viability ranged from 8.4 to 9 Log CFU.mL^−1^. The variation is part of the uncertainties of the study, considering that the freeze-dried culture was treated directly in both the passion fruit and the passion fruit with green tea-based media.

### 2.4. Fermentation Kinetics

For the fermentation kinetics study, the sample was distributed in 15 mL Falcon tubes which were incubated under controlled temperature conditions, without agitation, where the fermentation process took place for up to 48 h. In the first 24 h, an aliquot of 15 mL was withdrawn every 2 h, and later every 12 h; for analysis of cell concentration by the direct counting method in the Neubauer chamber, the pH was monitored by performing direct measurements with a potentiometer, titratable total acidity (method 942.15) [30] and soluble solids, determined using a portable refractometer. In the literature, a new method for determining soluble solids was presented with a less destructive proposal, i.e., the portable detector of soluble solids presented by Wenchuan et al. [31]. This new detector promises fast results without changing the fruit but, due to the availability and proven efficiency of the determination method using the refractometer, this was the methodology adopted for our analysis.

The direct counting method in the Neubauer chamber was used to determine the concentration of cells during fermentation. This method allows direct visualization and quick counting of the number of cells present in the medium. For cell counts, serial dilutions of up to 1:150,000 of the fermented medium in sterile distilled water were performed, and the count was performed using an ordinary light optical microscope. The result was expressed as Log CFU.mL^−1^.

Some parameters that describe the biological characteristics of the culture in the fermentation medium were calculated from the cell concentration data during the fermentation time (48 h). Such parameters were the maximum growth rate (µ_max_) and generation time (t_g_), calculated by Equations (1) and (2):

(1) $$\ln\left(\frac{X}{X_{i}}\right)=\mu_{\max}\left(t-t_{i}\right)$$

(2) $$\mu_{\max}=\frac{\ln2}{t_{g}}$$

where X is the concentration of cells at the end of the exponential phase (Log CFU.mL^−1^); X_i_ is the cell concentration at the beginning of the exponential phase (Log CFU.mL^−1^); t is the time corresponding to the end of the exponential phase (h); t_i_ is the time corresponding to the beginning of the exponential phase (h); t_g_ is the generation time (h); and µ_max_ is the maximum growth rate (h^−1^).

### 2.5. Statistical Analysis

The statistical significance of the independent variables was evaluated using analysis of variance (ANOVA) considering the value of *p* < 0.05 as significant. Linear regression analysis was used to adapt the mathematical model to the experimental data. The correlation coefficient R² was used to express the goodness of fit. Data collected using the full experimental design was analyzed using Statistica Software 7.0, Tulsa, OK, USA (www.statsoft.com) (accessed on 18 November 2021) [32]. The distribution of growth data under the optimal conditions of each fermentation medium was examined for normality testing and a correlation analysis was used to compare the mean scores between the evaluated groups, performed using the GraphPad Prisma version 8.0.0 for Windows, San Diego, CA, USA (www.graphpad.com) (accessed on 18 November 2021) [33].

## 3. Results and Discussion

### 3.1. L. gasseri Growth Curve during the Fermentation Period

**Figure 1:**
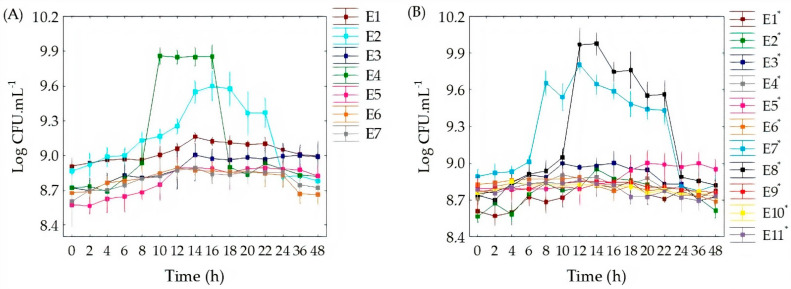
The concentration of L. gasseri cells during fermentation in passion fruit (**A**) and passion fruit added with green tea (**B**). * Test using passion fruit added with green tea.

The concentration of cells during fermentation of passion fruit and passion fruit added to green tea is shown in Figure 1. When analyzing Figure 1A, different durations of the latency phase can be observed in passion fruit juice, when the effect of different fermentation conditions is verified. The same can be observed when different concentrations of green tea are added to passion fruit juice (Figure 1B). The latency phase (lag phase) corresponds to the period of adaptation of the culture to the culture medium. In these and subsequent steps, the pH and incubation temperature are one of the most influential factors in the growth of probiotic cultures [34].

Passion fruit with pH adjusted to 7.5 and incubated at 44 °C resulted in a higher concentration of cells. In this assay, the latency phase lasted approximately 4 h, followed by a period of exponential growth, where an increase in cell concentration of more than 1 Log CFU.mL^−1^ was verified, reaching the maximum number of cells after 10 h of fermentation. This maximum production condition persisted until 16 h of fermentation, wherefrom that moment on the cell concentration reduced with time. Passion fruit with pH 3.5, close to the natural pH of the juice (2.7 ± 0.3) also showed good results, with high cell counts under the effect of the temperature of 44 °C, only inferior to the test with pH 7.5 and incubation temperature of 44 °C. The acidic pH of fruits such as passion fruit is one of the main challenges for the production of probiotic foods, as it influences the viable cell count; however, some strains have shown greater adaptation to acidic environments. The greatest effect of pH in our study can be seen in the data plotted in Figure 1A,B, where the bell-shaped curves are shifted especially as a function of pH.

In passion fruit with green tea, adjusting the pH to 7.5 was fundamental for the growth of *L. gasseri* at a temperature of 44 °C, where we observed a high count of probiotics. It is also noted when evaluating Figure 1B that the highest concentration of cells was obtained by adding 15% of green tea, followed by 7.5% of green tea. According to the results of this research, it can be assumed that the change in the composition of the medium with the addition of green tea was more influential than the pH for these conditions of study. Furthermore, the concentration of *L. gasseri* cells was reached in higher numbers in the fermentation processes with the added medium of green tea, reaching the maximum number of cells after 12 h of fermentation.

### 3.2. Biological Parameters of Growth Kinetics of L. gasseri in Passion Fruit and Passion Fruit Added to Green Tea

The maximum growth rate (µ_max_), generation time (t_g_), and maximum cell concentration (Log CFU.mL^−1^) reached in the fermentation process of the media using passion fruit and passion fruit with green tea were selected as the dependent variables to evaluate the effects of pH variation, incubation temperature (°C) and green tea concentration (%). The maximum rate of cell growth is related to generation time. In Table 2, for the culture medium using passion fruit, the maximum growth rate (0.28 h^−1^) was observed in the fermentation conditions that involved a medium pH of 7.5 and an incubation temperature of 44 °C. Under these conditions, a generation time of approximately 2.5 h was observed, where a maximum cell concentration of more than 9.8 Log CFU.mL^−1^ was reached.

**Figure 2:**
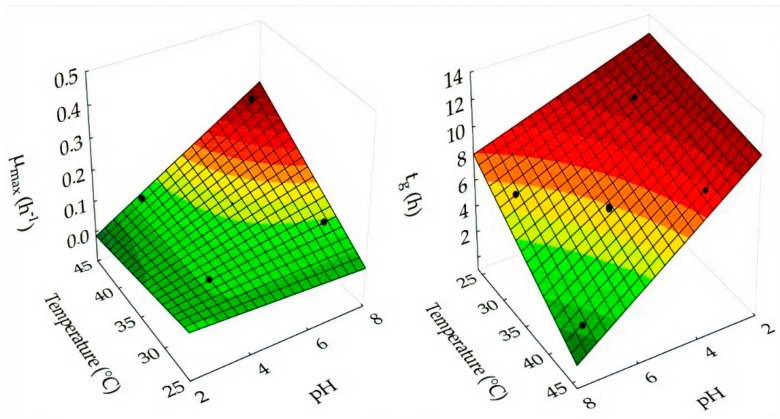
Effect of incubation pH and temperature on maximum growth rate and generation time of *L. gasseri* in passion fruit juice.

A polynomial model was fitted to the experimental data to evaluate the effects of fermentation conditions on media using passion fruit and passion fruit added to green tea. Response surfaces were constructed from Equations (3) and (4) to simulate the effects of pH and incubation temperature on the maximum growth rate and generation time of *L. gasseri* in passion fruit (Figure 2). The incubation pH and temperature did not significantly influence the value of the maximum cell concentration reached during fermentation.

(3) $$\mu_{\max}=0.297-0.078\times \mathrm{pH}-0.009\times T+0.003\times \mathrm{pH}\times T$$

(4) $$t_{g}=13.753+0.256\times \mathrm{pH}+0.017\times T-0.043\times \mathrm{pH}\times T$$

where the maximum growth rate (µ_max_) has R² = 0.9573 and the generation time (t_g_) has R² = 0.9877, pH represents the pH variable, and T the incubation temperature (°C).

In the medium using passion fruit with green tea, the maximum growth rate (0.19 h^−1^) was observed under the effect of pH 7.5, incubation temperature of 44 °C, and green tea concentration of 15%. It is noted when analyzing the data presented in Table 3 that the generation time under these conditions was approximately 3.7 h, reaching a maximum number of cells of approximately 10 Log CFU.mL^−1^.

**Figure 3:**
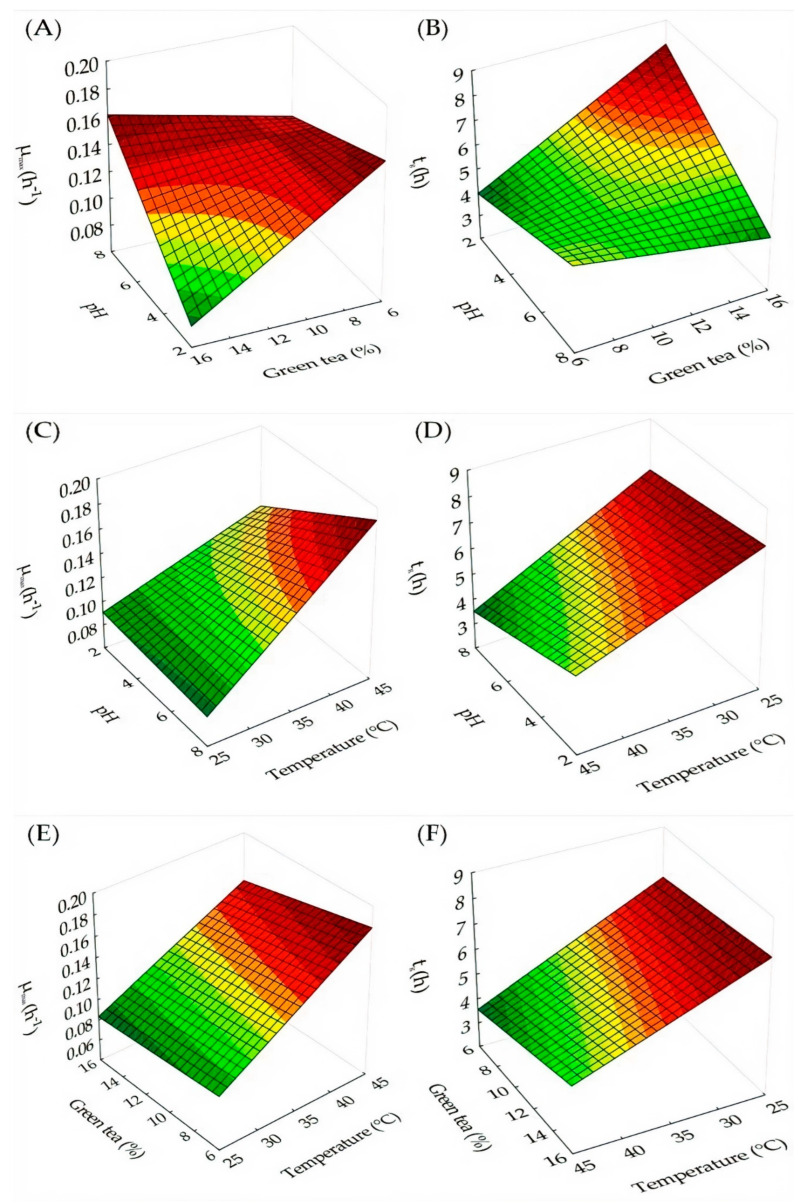
Effect of pH and green tea concentration on maximum growth rate (**A**) and generation time (**B**), pH and incubation temperature on maximum growth rate (**C**) and generation time (**D**), tea concentration green and incubation temperature on the maximum growth rate (**E**) and generation time (**F**) of *L. gasseri* in passion fruit juice.

**Figure 4:**
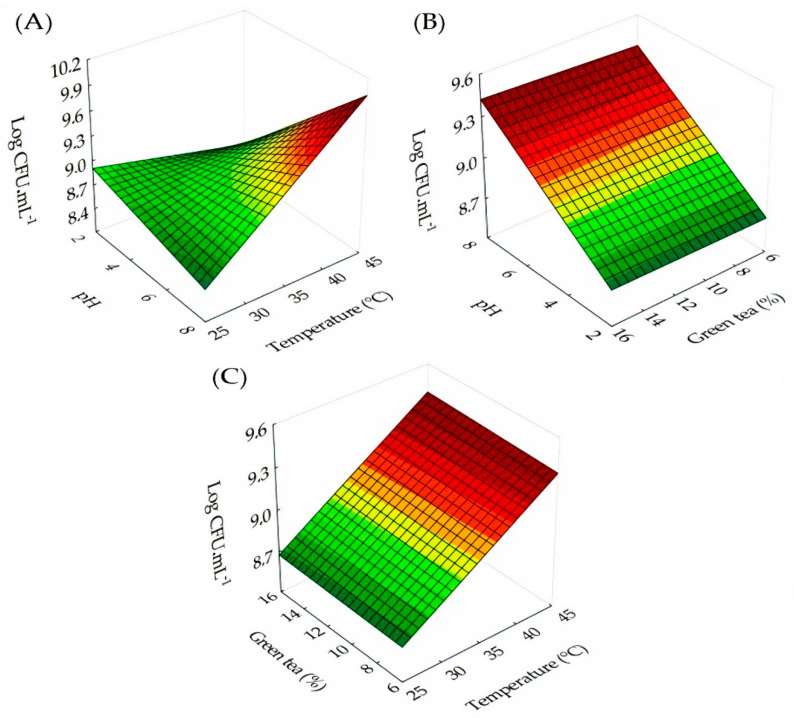
Effect of incubation pH and temperature (**A**), green tea pH and concentration (**B**), and green tea concentration and incubation temperature (**C**) on the maximum concentration of *L. gasseri* cells in passion fruit juice.

For the medium using passion fruit with green tea, the surface responses to the effects of pH, incubation temperature (°C), and green tea concentration (%) were constructed using Equations (5) and (6) for the rate of maximum growth (µ_max_) and generation time (t_g_), respectively. The effects of the independent variables are observed in Figure 3A,C,E for the maximum growth rate (µ_max_) and in Figure 3B,D,F for the generation time (t_g_). The pH and incubation temperature for the medium using passion fruit with green tea significantly influenced the value of the maximum cell concentration reached. The effect of these conditions was plotted in Figure 4 through Equation (7).

(5) $$\begin{array}{l} \mu_{\max}=0.258-0.052\times \mathrm{pH}-0.017\times \mathrm{GT}+0.001\times \mathrm{pH}\times T+0.003\times \mathrm{pH}\times \mathrm{GT}+0.0001\times T\\ \times \mathrm{GT}-0.00004\times \mathrm{pH}\times T\times \mathrm{GT} \end{array}$$

(6) $$\begin{array}{l} t_{g}=3.948+1.365\times \mathrm{pH}-0.110\times T+0.589\times GT-0.015\times \mathrm{pH}\times T-0.112\times \mathrm{pH}\times \mathrm{GT}\\ +0.0006\times \mathrm{pH}\times T\times \mathrm{GT} \end{array}$$

(7) $$\mathrm{Log}\;\mathrm{CFU}.{\mathrm{mL}}^{-1}=10.346-0.468\times \mathrm{pH}-0.052T+0.016\times \mathrm{pH}\times T+0.00002\times \mathrm{pH}\times T\times GT_{\;}$$

where the maximum growth rate (µ_max_) has R³ = 0.9644 and the generation time (t_g_) has R² = 0.9558, the maximum cell concentration (Log CFU.mL^−1^) has R² = 0.837, pH represents the pH variable, T an incubation temperature (°C) and GT the concentration of green tea (%).

Xu et al. [35] report that *L. gasseri* is a culture found in the human body that shows excellent growth at a temperature of 37 °C and a pH of 5.5. However, we found higher values for the growth of *L. gasseri* under temperature and pH conditions a little above what is commonly reported in the literature. Since in fermentation processes several factors can influence the performance of certain organisms, the composition of the medium, as well as the initial concentration of the inoculum and the static condition in the incubation, may have been reflected in the verified results. Śliżewska and Chlebicz-Wójcik [34] observed different maximum growth rates and generation times for selected *Lactobacillus* strains depending on the culture medium. This indicated that, in addition to the conditions and pH and temperature, the composition of the medium is fundamental to efficiency in the elaboration of potential probiotic foods.

The correlation analysis between the concentration of *L. gasseri* cells during the 48 h of fermentation in the fermentation media using passion fruit and passion fruit with green tea showed a significant correlation, with *p* = 0.0006 and r = 0.7989. Therefore, the observed results indicate that the addition of green tea contributed positively to the growth of *L. gasseri* in passion fruit juice. Green tea leaves have a high composition of bioactive compounds such as phenolic acids, flavonoids, tannins, alkaloids, and nutrients such as carbohydrates, proteins, and minerals [36]. A previous study found that green tea leaf polysaccharides could modulate gut microbiota composition [21]. Zhang et al. [22] showed that purple sweet potato anthocyanins induced the proliferation of *Bifidobacterium* and *Lactobacillus/enterococcus* spp. and induced the growth of some pathogenic bacteria. Therefore, our study indicates that the addition of green tea in the fermentation medium formulation induced the growth of *L. gasseri* in passion fruit juice, due to the increase of bioactive compounds in the fermentation medium.

### 3.3. pH, Acidity and Soluble Solids during Fermentation by L. gasseri in Passion Fruit and Passion Fruit with Green Tea

**Figure 5:**
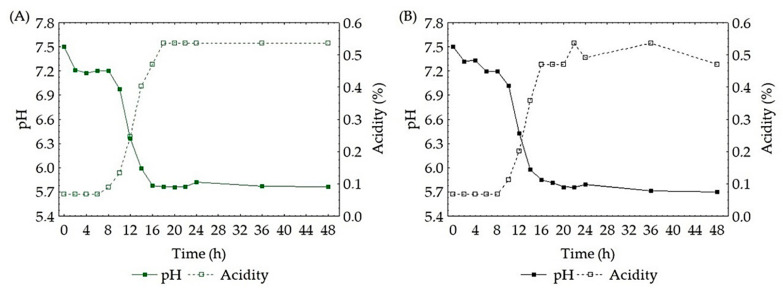
Monitoring pH of acidity in the best fermentation conditions of passion fruit juice (**A**) and passion fruit added with green tea (**B**).

During fermentation, pH, total acidity, and soluble solids were monitored for fermentation conditions using passion fruit (Figure S1) and passion fruit with green tea (Figure S2). The stress induced by the pH difference resulted in a decrease in the growth rate in the first hours of fermentation. Mousavi et al. [37] reported that the difference between the pH of the MRS broth used in the pre-cultivation, which has a pH of 5.6, and the initial pH of the pomegranate juice (3.09) resulted in decreased growth of cultures of *Lactobacillus paracasei*, *Lactobacillus acidophilus*, *Lactobacillus plantarum* and *Lactobacillus delbruekii* in the first 24 h. In curves with a high number of cells (E4: pH 7.5, the temperature of 44 °C and E8 *: pH 7.5, the temperature of 44 °C, green tea at a concentration of 15%) the pH decreased from 7.5 to 5.8, and from 7.5 to 5.7 in the fermentation media using passion fruit and passion fruit green tea, respectively. Fermentation conditions involving pHs 3.5 and 5.5 showed insignificant changes in numbers after 48 h of fermentation in both culture media. The same behavior can be observed in acidity monitoring. Non-significant changes in pH and acidity have already been reported for the fermentation process of passion fruit juice by *Lactobacillus reutei* [38], and in the fermentation of pomegranate juice for *L. paracasei*, *L. acidofillus*, *L. plantarum* and *L. delbruekii* [37]. In Figure 5, we observe the relationship between pH and acidity during fermentation by *L. gasseri* in the medium using passion fruit (Figure 5A) and in passion fruit with green tea (Figure 5B), produced under conditions that favored the high number of cells, maximum growth rate and shortest generation time.

**Figure 6:**
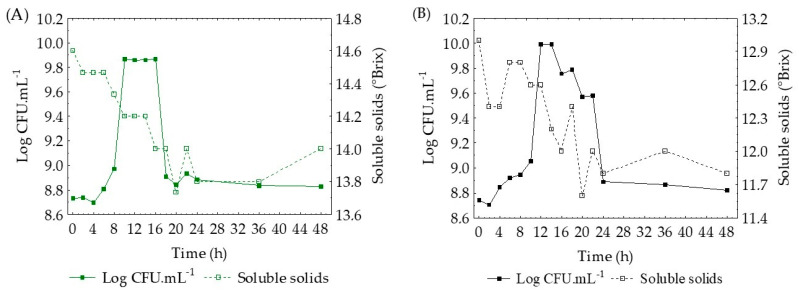
Monitoring of soluble solids with respect to cell concentration in the best fermentation conditions of passion fruit juice (**A**) and passion fruit with green tea (**B**).

*Lactobacillus* are nutritionally demanding organisms, with growth influenced by multiple factors such as formulation, pH, temperature, and inoculum concentration [18]. Soluble solids represent an important answer in the study of the kinetic characteristics of a fermentation process. Composed of sugars and organic acids, these parameter changes include the growth of lactic acid bacteria and the production of organic acids during the fermentation process. In Figure 6, we plot the relationship between the soluble solids content and the cell concentration over the 48 h of fermentation under conditions where we observed a high number of cells, maximum cell rate, and shorter generation time. During fermentation, the reduction of soluble solids showed a tendency to decrease with increasing cell concentration, for both fermentation media (passion fruit (Figure 6A) and passion fruit with green tea (Figure 6B), which indicates that the sugars were used for growth. However, in the tests with a lower growth rate, oscillations were observed, although insignificant. The soluble solids monitoring curves of all assays evaluated in the fermentation medium using passion fruit and passion fruit added with green tea were plotted in Figures S1C and S2C, respectively.

## 4. Conclusions

The addition of green tea to passion fruit boosted the growth of *L. gasseri,* which in turn improved the obtained product, adding functional components to the fermented product. The monitoring during the fermentation process of the media with passion fruit and passion fruit with green tea allowed discovery of the appropriate conditions for the production of the passion fruit-based probiotic food, comparing the different media and optimizing the process. Therefore, despite the superiority of the medium with green tea added, both formulations fermented by *L. gasseri* are promising products for consumption as functional food and for application in the food industry, with potential health benefits. However, during fermentation, changes in the medium can be observed; thus, other studies need to be carried out to understand the compounds formed during fermentation and to influence the quality of the product obtained.

## Figures and Tables

**Table 1:** Assay formulations based on full experimental design.

Passion Fruit			Passion Fruit Added to Green Tea			
Essay	pH	T (°C)	Essay	pH	T (°C)	Green Tea (%)
E1	3.5	30.0	E1 *	3.5	30.0	7.5
E2	3.5	44.0	E2 *	3.5	30.0	15.0
E3	7.5	30.0	E3 *	3.5	44.0	7.5
E4	7.5	44.0	E4 *	3.5	44.0	15.0
E5	5.5	37.0	E5 *	7.5	30.0	7.5
E6	5.5	37.0	E6 *	7.5	30.0	15.0
E7	5.5	37.0	E7 *	7.5	44.0	7.5
			E8 *	7.5	44.0	15.0
			E9 *	5.5	37.0	11.3
			E10 *	5.5	37.0	11.3
			E11 *	5.5	37.0	11.3

* Test using passion fruit added with green tea.

**Table 2:** Biological characteristics of *L. gasseri* in passion fruit juice fermentation.

Essay	pH	T (°C)	Parameters		
			µ_max_ (h^−1^)	t_g_ (h)	Log CFU.mL^−1^
E1	3.5	30.0	0.0637	10.8765	9.1612
E2	3.5	44.0	0.0769	9.0107	9.6213
E3	7.5	30.0	0.1028	6.7451	8.9831
E4	7.5	44.0	0.2802	2.4740	9.8685
E5	5.5	37.0	0.1039	6.6703	8.9075
E6	5.5	37.0	0.1023	6.7728	8.8983
E7	5.5	37.0	0.1020	6.7973	8.9055

**Table 3:** Biological characteristics of *L. gasseri* in the fermentation of passion fruit juice added with green tea.

Essay	pH	T (°C)	Green Tea (%)	Parameters		
				µ_max_ (h^−1^)	t_g_ (h)	Log CFU.mL^−1^
E1 *	3.5	30.0	7.5	0.1285	5.3929	8.8585
E2 *	3.5	30.0	15.0	0.0861	8.0506	8.9623
E3 *	3.5	44.0	7.5	0.1700	4.0773	9.0173
E4 *	3.5	44.0	15.0	0.1263	5.4890	8.9085
E5 *	7.5	30.0	7.5	0.1021	6.7854	9.0181
E6 *	7.5	30.0	15.0	0.1254	5.5284	8.8921
E7 *	7.5	44.0	7.5	0.1813	3.8235	9.8108
E8 *	7.5	44.0	15.0	0.1867	3.7124	9.9932
E9 *	5.5	37.0	11.3	0.1256	5.5168	8.8573
E10 *	5.5	37.0	11.3	0.1247	5.5570	8.8400
E11 *	5.5	37.0	11.3	0.1259	5.5034	8.8674

* Test using passion fruit added with green tea.

## Data Availability

Not applicable.

## Supplementary Materials

The following supporting information can be downloaded at: https://www.mdpi.com/article/10.3390/foods11101471/s1, Figure S1. Monitoring of pH (A), total titratable purity (B) and soluble solids (C) during fermentation by *L. gasseri* in passion fruit juice; Figure S2. Monitoring of pH (A), total titratable acidity (B) and soluble solids (C) during fermentation by *L. gasseri* in passion fruit juice.
