# Supplementary material for: Study of Fermentation Strategies by Lactobacillus gasseri for the Production of Probiotic Food Using Passion Fruit Juice Combined with Green Tea as Raw Material
Source: Foods. 2022 May 18;11(10):1471. doi: 10.3390/foods11101471 (PMC9141917; doi:10.3390/foods11101471)

Figure S1. Monitoring of pH (A), total titratable purity (B) and soluble solids (C) during fermentation by *L. gasseri* in passion fruit juice.

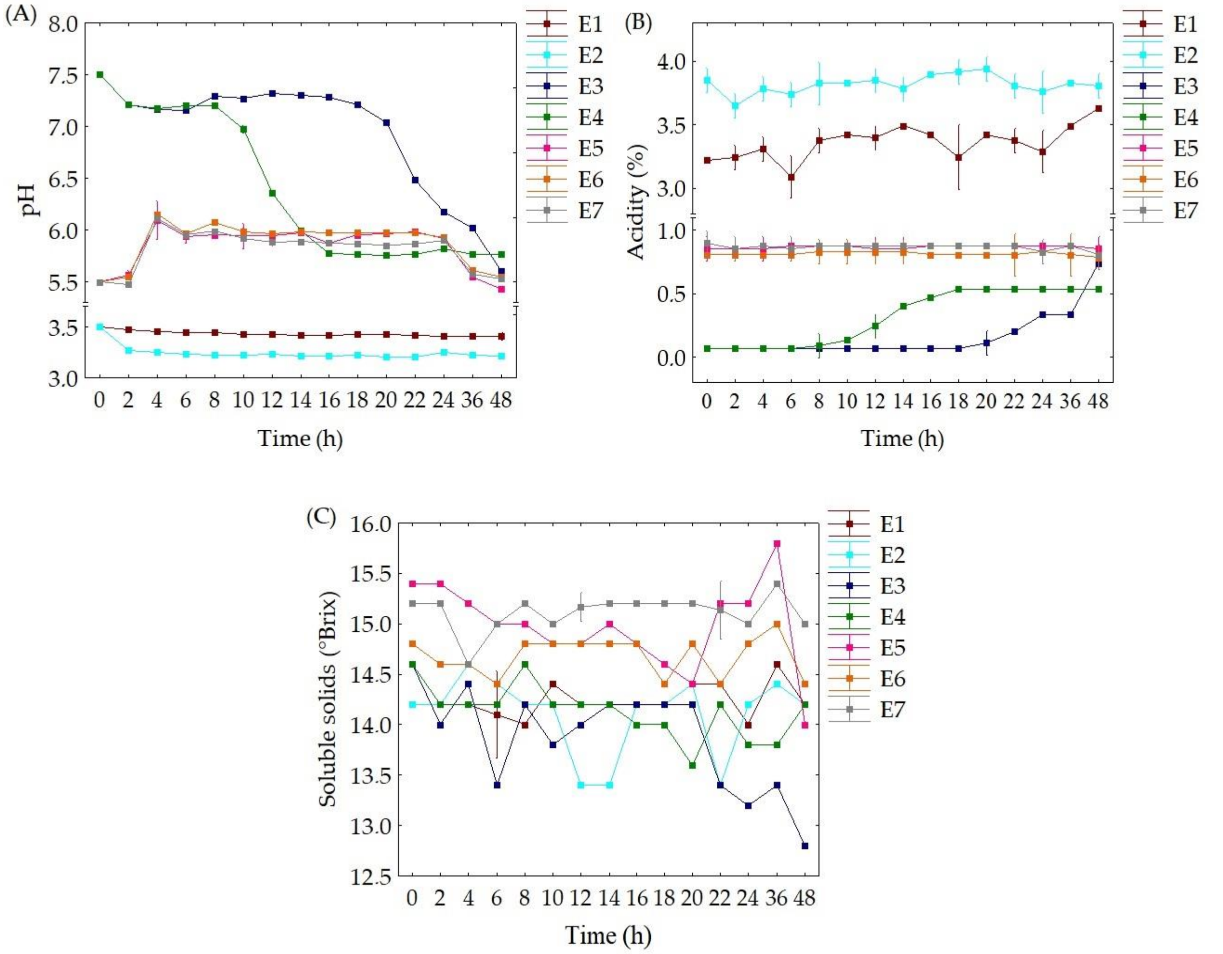

Figure S2. Monitoring of pH (A), total titratable acidity (B) and soluble solids (C) during fermentation by *L. gasseri* in passion fruit juice.

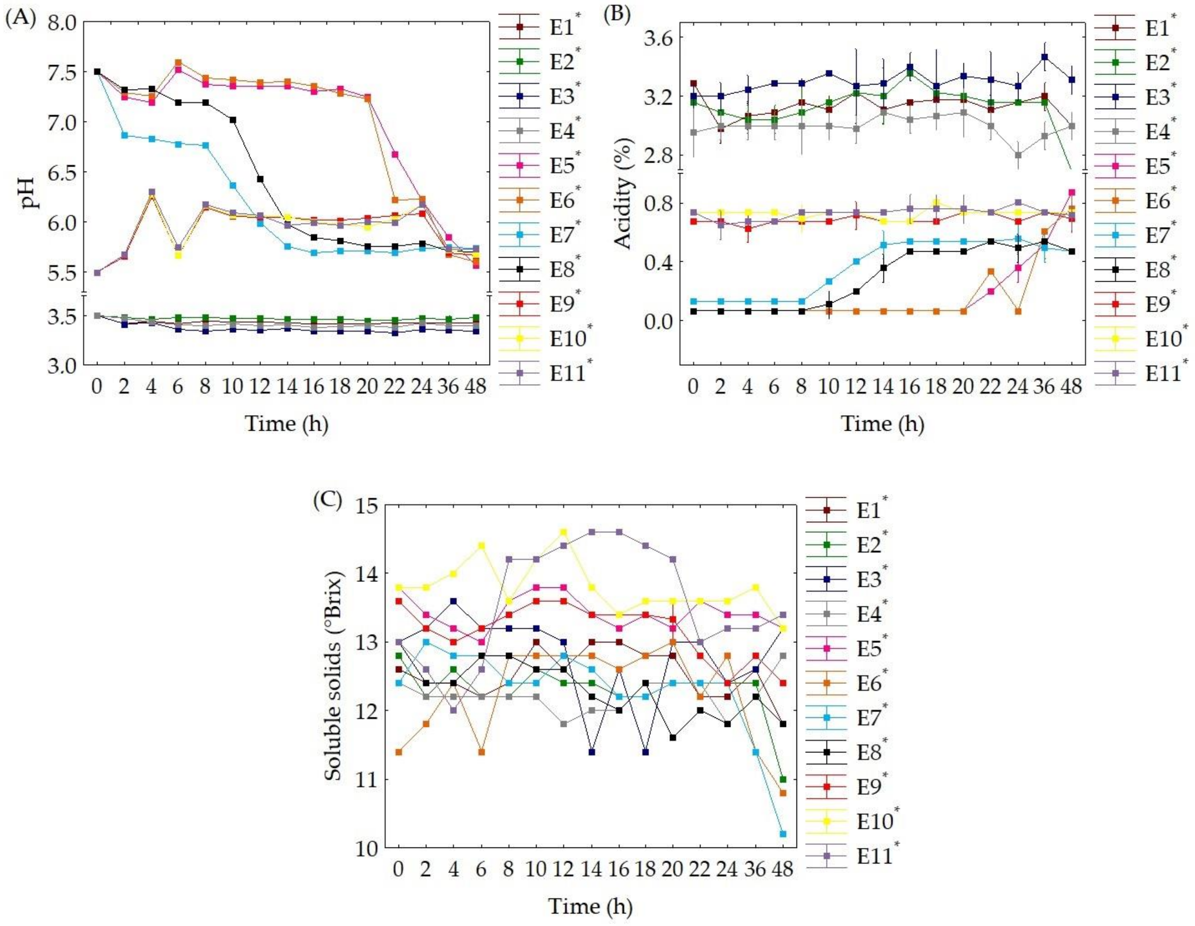

Supplement: Supplementary file 1 [file foods-11-01471-s001.zip › foods-1722093-supplementary.pdf]
